# Moral Disengagement in Youth Athletes: A Narrative Review

**DOI:** 10.3390/jfmk7020033

**Published:** 2022-04-15

**Authors:** Ambra Gentile, Ivana Milovanovic, Saša Pišot, Antonino Bianco, Gioacchino Lavanco

**Affiliations:** 1 Department of Psychology, Educational Sciences and Human Movement, University of Palermo, 90133 Palermo, Italy; antonino.bianco@unipa.it (A.B.); gioacchino.lavanco@unipa.it (G.L.); 2 Faculty of Sport and Physical Education, University of Novi Sad, 21000 Novi Sad, Serbia; ivana.milovanovic@uns.ac.rs; 3 Institute for Kinesiology Research, Science and Research Centre Koper, 6000 Koper, Slovenia; sasa.pisot@zrs-kp.si

**Keywords:** moral disengagement, aggression, cheating, physical activity, moral development, children, adolescence

## Abstract

The sports environment can be considered as a context characterized by interactions typical of social groups, where children have the chance to learn good values. Positive and negative behaviours in sports, also called prosocial and antisocial behaviours, have been studied according to a moral perspective, as has doping behaviour, taking into consideration the concept of moral disengagement. Moral disengagement in children has been associated with maladaptive behaviours later in life, even though it should disappear with growth. Concerning the sports environment, previous reviews on the topic have extensively illustrated the role of moral variables in sport and their relation to antisocial behaviour and doping, positing some research questions that should be investigated in the future. Starting from these questions, the current narrative review aims to update literature about the effects of moral disengagement on youth athletes. Therefore, new studies about the predictors of moral disengagement are introduced, followed by contributions concerning the relationship between moral disengagement and doping and between moral disengagement and antisocial behaviour. Finally, the review summarizes which research questions have been solved in the last decade and which should be researched further on.

## 1. Introduction

Sports can be considered as a social context characterized by interactions and reciprocal influence among the actors. These interactions can produce positive consequences for others, such as prosocial behaviour, discipline, and honesty, but also negative actions, like cheating, intimidating, and injuring other participants [1]. Studies concerning children and youth in sports have pointed out the role of moral reasoning as a determinant of prosocial and antisocial behaviour [2]. The degree to which children act prosocially depends on their achievement goal orientation [2,3,4], while aggressive tendencies and antisocial behaviour are predicted by a less mature moral reasoning [5,6] and a high level of moral disengagement.

Moral disengagement is a set of psychological mechanisms used to disengage transgressive behaviour from the self-sanctions that keep behaviour in line with moral standards [2,7]. These mechanisms consist of eight cognitive strategies converting a transgressive behaviour into an acceptable one to keep the behaviour in line with moral standards [8]. The strategies are: moral justification, euphemistic labelling, advantageous comparison, displacement of responsibility, diffusion of responsibility, distortion of consequences, dehumanization, and attribution of blame [9].

The concept of moral disengagement has been identified as an explanation for several disruptive behaviours, such as drug abuse and delinquency [10]. For what concerns children and adolescents, it was associated with aggression and rule-breaking behaviour [11]. Longitudinal studies showed that this tendency usually disappears with growth, but 10% of the population maintains it also in adult life [12].

Bandura, Barbaranelli, Caprara and Pastorelli [9] suggested that moral disengagement is a context-specific phenomenon and should be separately analysed. This is particularly true if we consider that when it comes to youth, moral reasoning—i.e., the criteria that people use to resolve moral conflicts—shows differences between sports and daily life. This phenomenon is well-known as “bracketed morality” [13]. Moreover, certain conditions existing in sports may facilitate moral disengagement [14].

Concerning the sports field, in the last decades moral disengagement has been extensively studied in relation to antisocial and aggressive behaviour [15,16,17] and doping [18,19]. Several reviews have pointed out the mechanisms of moral disengagement [11,20,21,22,23], and the latest contribution in the sports context was in 2011 [24], where the authors reported the need for further research in the role of regulatory emotions such as anticipatory guilt [9]. Therefore, the present descriptive review aims to update the scientific literature analysing the role of moral disengagement in youth athletes’ behaviour, focusing on predictors of moral disengagement in sports and referring to doping and antisocial behaviours as outcomes. The present review considers studies published between 2012 and 2022 with children, adolescents, and young adults as the target group, considered under the label “youth.”

## 2. Predictors of Moral Disengagement in Sports

Morality is a strong component of sport participation. A number of studies have considered morality as a good predictor of antisocial behaviour [4,25,26,27,28]. Specifically, moral disengagement has been identified as the key predictor of antisocial behaviour. The questionnaire Moral Disengagement in Sport Scale (MDSS) by Boardley and Kavussanu [29] reported a strong positive correlation with self-reported antisocial behaviour, especially in boys. Given the cruciality of the topic for youth development, it also becomes important to point out what the predictors are.

Shields et al. [30] hypothesized some factors that could predict moral disengagement in sports. Several studies reported gender as a significant predictor of moral disengagement, where boys tended to disengage to a bigger extent than girls, also in the sports context [15,17], and the authors confirmed this result. Age is also revealed to be a significant predictor of moral disengagement, with a stronger relationship between moral disengagement and bullying in adolescents than in children, regardless of their involvement in sports activities [31].

Another predictor of moral disengagement, identified by Shields, Funk, and Bredemeier, is moral identity, which refers to the cognition people hold when thinking about their moral character and their desire to be moral persons [32,33]. The authors found that, after gender, moral identity was the strongest predictor of moral disengagement. In other words, the desire to be a moral person negatively predicts the likelihood of morally disengaging from an antisocial action. Furthermore, moral attentiveness, which relates to the importance that individuals give to morality in their experiences [34], was a significant predictor. Finally, contesting orientation, which is the metaphorical framework used to understand the values and meaning of contesting [35], was considered a predictor. The authors found that a contesting orientation focused on “partnership” orientation, where contesting is seen as a mutually beneficial process of enjoyment and growth, negatively predicted moral disengagement. In contrast, a contesting orientation of “war,” which is metaphorically meant as a battle allowing only one winner, positively predicted moral disengagement. Empathy did not predict moral disengagement in sports.

Budziszewski et al. [36] found similar results in a sample of athlete trainers, whose warfare–contesting orientation, partnership–contesting orientation, sports ethic, commitment, and social identity were significantly related to moral disengagement. Specifically, athlete trainers reporting a stronger sports ethic and contesting orientations with lower commitment and social connection to student–athletes were more prone to moral disengagement, allowing athletes to play through injury.

Another study by Jones et al. [37] found that narcissism predicts moral disengagement and antisocial behaviour within the sports context. The authors hypothesized that, since narcissism is negatively related to empathy and positively related to the feeling of entitlement, narcissists should be more likely to disengage morally and to behave antisocially, and their result seems to support this hypothesis. Indeed, typical traits of narcissists are manipulation and antisocial personality [38,39], together with the desire for success [40]. In line with this hypothesis, narcissists may think that moral standards are not valid in the sporting context, transforming antisocial behaviour into a morally acceptable action [41].

Finally, Caz et al. [42] hypothesized that athletic identity could be a predictor of moral disengagement associated with doping. Brewer et al. [43] identified three features of athletic identity, namely social identity, exclusivity, and negative affectivity. Social identity focuses on the extent to which the athletes view themselves as athletes in other people’s eyes [44]. Exclusivity is the degree to which the athlete assumes a self-concept related only to the athletic image. Negative affectivity is the extent to which the athlete worries about poor performance in the game or not being able to fulfill the athletic role. The study of Caz found that athletes’ perception of social identity has a positive effect on moral disengagement related to doping, while exclusivity was negatively related to it. Negative affectivity did not have any relation with moral disengagement. According to the authors, the social identity perceptions of the athletes trigger the tendency towards moral disengagement associated with doping. Moreover, concerning exclusivity, when sports become the unique goal in athletes’ lives and success becomes of secondary importance, the likelihood of using moral disengagement mechanisms concerning doping decreases.

## 3. Moral Disengagement as a Predictor of Doping Behaviour

The use of performance enhancement drugs to improve athletic performance is a serious concern in sports (see Table 1). Understanding the mechanisms behind doping becomes critical because previous studies have highlighted a worrisome tendency towards doping in adolescent athletes [45,46,47,48,49].

First of all, enhancing performance through illicit substances may have adverse health effects at the cardiovascular, psychiatric, endocrine, neurologic, hepatic, renal, or musculoskeletal levels, but these negative consequences are often underestimated [58]. Moreover, apart from the health risks, doping is a moral issue [59,60]. Framing doping as a moral problem allows one to examine the influence of morality on the likelihood of engaging in doping behaviour [19]. Barkoukis and Elbe [61] analysed both the moral and ethical implications of doping, concluding that morality has been mainly framed in a socio-cognitive approach. In contrast, the ethical aspect has been considered to a less extent. Two qualitative studies by Boardley et al. [56,57] reported that athletes who previously had used performance enhancement drugs tend to use from six to seven moral disengagement mechanisms. Another study of Drewery and Wilson [55] specifically found that displacement of responsibility and advantageous comparison significantly predict attitudes towards doping, especially in athletes with obsessive passion. 

A review of Kavussanu [62] deeply examined the process of moral disengagement occurring in doping in the sports field, taking into account the theory of planned behaviour [63,64], the self-determination theory (SDT) [65], and the social cognitive theory [7].

According to the theory of planned behaviour, the best predictor of action is not the attitude towards the targeted behaviour, but the intention of engaging in it [64]. Lucidi, Grano, Leone, Lombardo and Pesce [63] employed this framework to detect doping by asking the intention to use illegal substances to improve sports performance and physical appearance, together with a questionnaire of moral disengagement related to doping [66]. After three months, the researchers asked the sample to indicate which doping substances they used during the past three months to improve their athletic performance and/or physical appearance. The results showed a positive prediction of moral disengagement towards the intention to dope and the later doping engagement, and higher scores in moral disengagement were related to more positive attitudes towards doping. Moreover, participants with a high level of moral disengagement tended to consider important the approval of others and were less likely to withdraw from doping if there was the chance. The real limitation of the study concerned the participating sample, which consisted of athletes who did not regularly compete in sports.

In recent times, the intention to engage in doping was related to moral disengagement, also considering the motivational climate. One of the possible understandings of motivational climate in sports is the distinction between a task-involving climate, where the athletes are provided with a clear rationale for tasks and non-controlling competence feedback, and ego-involving climate, where competence feedback is provided and the athletes define success as outperforming others and winning [67]. A study by Guo, Liang, Baker and Mao [19] examined the association among perceived motivational climate (i.e., task-involving and ego-involving), moral variables (i.e., moral disengagement and sportspersonship), and attitudes towards doping with doping intention. The results showed that the task-involving motivational climate was negatively associated with doping intention through sportspersonship, while the ego-involving climate was positively related to doping intention through moral disengagement. Therefore, athletes not receiving feedback on their competencies reported higher levels of sportspersonship and a lower likelihood to engage in doping, while athletes receiving feedback on their competencies were more likely to morally disengage from their actions and reported higher intention to engage in doping behaviour.

Moral disengagement has also been related to the self-determination theory (SDT) [65], particularly controlled motivation, which occurs when individuals join sports for extrinsic rewards associated with sports participation. Following this perspective, doping is seen as the mean of allowing the athletes to achieve their goals [62]. A study by Hodge, Hargreaves, Gerrard and Lonsdale [18] reported a correlation between controlled motivation and moral disengagement, meaning that athletes competing in sports for extrinsic reasons are at risk of moral disengagement. However, the correlation between moral disengagement and controlled motivation was low, suggesting that SDT might not be the best framework for explaining the engagement in doping.

Another study focused on the influence of coaching style on athletes’ moral disengagement [54]. Coaches usually play a fundamental role in shaping athletic performance and relative psychological experience [68]. Coaching style, according to the SDT framework, can be divided into autonomy-supportive and controlling styles [69]. A coach who follows the autonomy-supportive style gives athletes the opportunity to contribute to decision-making, is attentive to their views and feelings, and allows them to choose appropriate tactics or techniques. Conversely, a controlling coaching style is characterized by an authoritarian and coercive manner, guilt induction strategies, manipulation, threats, and a lack of recognition of the athletes’ perspectives and feelings. The study of Chen, Wang, Wang and Huang [54] found a relationship between the controlling style of coaching and attitudes towards doping and the mediation of moral disengagement. This means that a controlling coaching style predicts the probability of moral disengagement, which, in turn, produces a positive attitude towards doping.

The social-cognitive theory has been used as a framework for explaining moral disengagement in sports through the concept of anticipated guilt [7]. After acting out a transgressive behaviour, guilt is an adaptive emotion that prevents people from repeating the same action that moral disengagement can reduce, thus facilitating doping [62].

A study of Ring and Hurst [52] examined the effects of moral disengagement on the likelihood of doping, with a mediation of guilt and a moderation of moral traits. In this study, the anticipated guilt, that is, the emotion preventing feelings of guilt, was related to six moral scenarios corresponding to the six moral disengagement mechanisms in sports. The findings indicated that the likelihood of doping was higher in all the six mechanisms compared to the neutral scenario. Moreover, anticipated guilt mediated the relationships between five mechanisms (excepted from euphemistic labeling) and doping likelihood. Finally, the effect of moral disengagement on the likelihood of doping was moderated by moral agency, moral perfectionism, and moral values. These results indicated that moral disengagement increases the likelihood of engaging in doping by decreasing affective self-sanction for doping [52]. Similar results were also reported by Kavussanu and Ring [53], Harris et al. [50], Ring and Kavussanu [48], and Stanger and Backhouse [51], where the anticipated guilt about potential doping mediated the relationship between moral disengagement and the likelihood of doping.

## 4. Moral Disengagement as a Predictor of Antisocial Behaviour

In past decades, a high number of studies have connected moral disengagement to antisocial behaviour in the practice of sports (see Table 2). Antisocial behaviour in sports is defined as a negative, intentional, motivated social action directed to opponents and/or teammates, such as provoking, pushing, and being aggressive or violent [70]. Traditionally, moral disengagement has been considered a predictor of antisocial behaviour, but Kavussanu and Stanger [71] hypothesized that it could be equally plausible to consider it as an outcome. As maintained by the authors, this relation can be thought of as bidirectional.

Stanger et al. [76] investigated the effect of moral disengagement mechanisms on emotions and antisocial behaviour towards opponents by first analysing the mediating role of anticipated guilt and then investigating the role of attribution of blame, which is a mechanism of moral disengagement. Specifically, in the second study, the authors administered a picture-viewing task, where athletes are required to rate images depicting antisocial behaviour situations (e.g., a rugby player who had “fouled” and had hurt another player). Afterward, participants in the experimental group read that the victim’s injurious behaviour was deliberate and that he had mocked the perpetrator. In this way, the participants were forced to believe that the perpetrators were retaliating after being “fouled” by the victim. In the control group, participants did not receive any instruction. The findings from the first study reported a positive correlation between moral disengagement and antisocial behaviour, with a partial mediation of anticipated guilt. In other words, athletes were more likely to act antisocially when they did not experience anticipated guilt. From the second study, the authors reported that the attribution-of-blame mechanism decreased the negative emotional reaction to antisocial behaviour, increasing the likelihood of antisocial behaviour.

In a longitudinal perspective, a recent study by Boardley et al. [73] examined the relationship between moral disengagement and antisocial behaviour in sports. The authors found that earlier antisocial behaviour was a strong positive predictor of later antisocial behaviour and that earlier moral development was related to later moral development. Moreover, moral development predicted longitudinal changes in antisocial behaviour towards opponents.

Recent studies also considered the role of the group in the investigation of moral disengagement. Danioni et al. [72], referring to a recent study of Gini, Pozzoli and Bussey [31], posited that interpersonal and social factors should be considered when investigating moral disengagement. According to Bandura [79], collective moral disengagement arises from interactive, coordinative, and synergistic group dynamics when justifying negative actions within a significant social group and can contribute to the development of group norms and of behaviours, including the same mechanisms as those of individual moral disengagement. Therefore, collective moral disengagement might be a significant construct for sports teams, particularly for adolescents. In Danioni’s study, collective moral disengagement significantly predicted antisocial behaviour towards teammates and opponents. The most interesting finding was that a performance-oriented climate moderated this relationship. In other words, when the climate was performance-oriented, collective moral disengagement was a predictor of antisocial behaviour.

Other studies have related moral disengagement to the team motivational climate in antisocial behaviours [16,75,80]. As mentioned before, motivational climate can be referred to goal orientations (i.e., ego-orientation vs. task-orientation) that interact with social climate factors [81]. Concerning social climate factors, Ames [82] distinguished between mastery (task) and performance (ego) perceptions of motivational climates. When students are involved in a decision-making task whose success depends on the efforts of all the members, it is more likely that the students will perceive their classroom as mastery-oriented [83]. Conversely, students will perceive the climate as performance-oriented when they are evaluated by normative standards and when the interpersonal comparison is cued. Ames and Archer [83] argued that this latter motivational climate increases the probability of observing maladaptive motivational responses in students. Concerning moral disengagement and antisocial behaviour, Stanger, Backhouse, Jennings and McKenna [16] found that mastery climate was negatively associated with prosocial behaviour towards teammates both directly and indirectly via social support and that the prosocial opponent behaviour was indirectly and positively associated via social support and perspective-taking. Moreover, mastery climate was negatively associated with antisocial behaviours towards teammates and opponents indirectly via social support, perspective-taking, and moral disengagement. Finally, concerning antisocial behaviours, moral disengagement directly and indirectly moderated the relationship between antisocial behaviours towards both teammates and opponents. These findings suggest that in adopting a mastery climate and avoiding overemphasis on a performance climate, the likelihood of adopting antisocial behaviour notably decreases.

Bruner et al. [78] examined moral disengagement in a social identity theory (SIT) perspective [84], focusing on two out of the three key dimensions of social identity: ingroup ties and ingroup affect. “Ingroup ties” refers to the perception of similarity, bonding, and belongingness with other group members, while “ingroup affect” relates to the positive feeling associated with group membership [85]. In the study of Bruner, ingroup ties and ingroup affect were related to moral disengagement and to prosocial and antisocial behaviour. The results showed that overall ingroup affect has a positive effect on prosocial behaviour towards teammates and negative effects on antisocial behaviour toward teammates and opponents. Furthermore, all effects are mediated by moral disengagement. Overall ingroup ties have a positive effect on prosocial behaviour towards teammates and antisocial behaviour towards teammates and opponents; no effects of ingroup ties on the three types of behaviour are mediated by moral disengagement. Finally, no dimension of social identity influenced prosocial behaviour towards opponents.

Boardley and Kavussanu [24] maintained that moral disengagement mechanisms should be related to specific antisocial behaviours, such as cheating and aggression. Concerning cheating, a study of Šukys [77] specifically investigated the relationship between moral disengagement and cheating behaviour by analysing the justification of deception. The justification was related to the manipulation of the results of the sports contest, to the manipulation of the rules of the sport contest, and to provocative behaviour towards opponents. Moreover, the study also considered the respondents’ years of experience. The results showed that moral disengagement is a predictor of the general justification of cheating in sports. Deceptive actions associated with athletes’ manipulation of the rules of the sports contest were justified to a larger extent by athletes with more experience in sports and by younger athletes. Regarding aggression, youth playing contact sports tended to report higher levels of moral disengagement than athletes in non-contact sports and a higher perception of legitimacy of aggressive behaviour in competition [74]. The study also confirmed the existence of age differences in moral disengagement, where younger athletes (17–18 years) tended to report higher levels than older athletes. 

## 5. Conclusions

Moral disengagement theory appeared almost thirty years ago, but its applications to daily life and sports remain still valid. As regards sports, moral disengagement has been identified as one of the main determinants of negative behaviours such as doping and of antisocial behaviours. Starting from the review of Boardley and Kavussanu [24], the main goal of the present descriptive review was to provide an update from the last ten years about the investigation of moral disengagement in youth athletes.

Some research questions that have been raised in previous studies, such as cheating and aggression behaviour and the role of regulatory emotions (anticipatory guilt), were answered through various studies. Specifically, in the first case, several studies highlighted the mediational role of anticipatory guilt in the relation between moral disengagement and antisocial behaviour/doping intention. Concerning the predictors of moral disengagement in sports, few studies were found in the last decade. Considering doping behaviour, the research mainly focused on self-regulatory mechanisms such as anticipated guilt and on motivational climate, athletic identity, moral identity, moral traits, and coaching style. Antisocial behaviour research focused on the role of motivational climate, locus of control, negative emotions, and social identity.

Despite the progress reached in these years, some research questions still remained unsolved, such as the influence of fear of failure on the relationship between moral disengagement and antisocial behaviour [30] or the use of a research design other than the cross-sectional or longitudinal that could offer a different perspective on these relationships [24]. Furthermore, it would be useful to analyse potential age differences in anticipatory guilt.

Future research should focus on these questions and provide new insights for the prevention of negative behaviours in the field of sports. Since sports activity has a strong impact on children and adolescents’ growth, being aware of this process may help reduce antisocial and doping behaviours during growth and adulthood in favour of a “clean sports” message.

## Figures and Tables

**Table 1:** Studies on moral disengagement as predictor of doping behaviour published between 2012 and 2022.

Authors	Year	Variables	Results
Harris, Smith and Myers [50]	2021	Moral disengagement Anticipated Guilt Consideration of doping	Anticipated guilt mediates the relation between moral disengagement and consideration of doping. Doping moral disengagement is a dominant predictor of doping-related cognitions, even when controlling for norms characterising the sport context.
Guo, Liang, Baker and Mao [19]	2021	Moral disengagement Perceived Motivational Climate Doping intention	Moral disengagement is positively associated with doping intention with a large effect size. Sportspersonship prevents doping intention. A task-involving motivational climate had a significant effect on doping intention. An ego-involving motivational climate was also found to be significantly associated with doping intention and wasfully mediated by moral disengagement.
Caz, Kayhan, Bardakci and Hasaan [42]	2021	Moral disengagement Athletic identity Doping	Athletes’ perception of social identity has a positive effect on doping moral disengagement, while the exclusivity was negatively related to it. Negative affectivity had no relation with moral disengagement.
Stanger and Backhouse [51]	2020	Moral disengagement Moral identity Doping Anticipated guilt	Dispositional moral identity is negatively associated with doping intention. Moral disengagement was positively associated with doping intention. Moreover, moral disengagement increases doping likelihood via anticipated guilt. Dispositional moral identity and inducing moral identity are linked with lower doping likelihood and attenuate the relationship between moral disengagement and doping likelihood. When the opportunities to morally disengage are amplified, the preventive effect of moral identity disappears.
Ring and Hurst [52]	2019	Moral disengagement mechanisms Doping likelihood Anticipated Guilt Moral traits	Doping likelihood is higher in all the six mechanisms compared to the neutral scenario. Moreover, anticipated guilt mediates the relationships between five mechanisms (excepted from euphemistic labeling) and doping likelihood. Finally, the effect of moral disengagement on doping likelihood is moderated by moral agency, moral perfectionism, and moral values.
Ring and Kavussanu [48]	2018	Self-regulatory efficacy Moral disengagement Guilt Doping likelihood	Doping self-regulatory efficacy is associated with doping likelihood both directly and indirectly through doping moral disengagement. Moral disengagement also contributes directly to higher doping likelihood and lower anticipated guilt about doping, which is associated with higher doping likelihood.
Kavussanu and Ring [53]	2017	Moral identity Doping likelihood Moral disengagement Anticipated guilt	Moral identity predicts doping likelihood indirectly via moral disengagement and anticipated guilt. Anticipated guilt about potential doping mediates the relationship between moral disengagement and doping likelihood.
Chen, Wang, Wang and Huang [54]	2017	Coaching style Attitude towards doping Moral disengagement	Controlling coaching style is positively associated with attitudes towards doping among Chinese athletes. The relationship is mediated by moral disengagement. An autonomy-supportive coaching style is not associated with attitudes towards doping.
Drewery and Wilson [55]	2016	Moral disengagement Passion Attitudes towards doping	Non-responsibility and advantageous comparison are significant predictors of attitudes towards doping. Obsessive passion mediates the influence of these factors on attitudes towards doping, while harmonious passion does not relate with them.
Boardley, Grix and Harkin [56]	2014	Moral disengagement mechanisms Doping behaviour	In 12 interviews, seven out of eight moral disengagement mechanisms were employed from athletes who had used performance enhancement drugs within the previous two years.
Boardley, Grix and Dewar [57]	2014	Moral disengagement mechanisms Doping behaviour	In 45 interviews, six out of eight moral disengagement mechanisms were employed by bodybuilders who had used performance enhancement drugs in the previous three months.
Lucidi, Zelli and Mallia [49]	2013	Moral disengagement Positive attitudes Self-regulatory efficacy to resist social pressure for doping Doping	Moral disengagement influences adolescents’ doping intention and doping use. From a longitudinal point of view, the more adolescents considered doping behaviour as acceptable, the more they showed positive attitudes towards it. Finally, adolescents with higher levels of doping moral disengagement expressed less personal self-regulatory efficacy.
Hodge, Hargreaves, Gerrard and Lonsdale [18]	2013	Doping Attitudes Motivation Moral disengagement	Moral disengagement strongly predicts positive attitudes towards doping, which, in turn, is a strong predictor of doping susceptibility.

**Table 2:** Studies on moral disengagement as predictor of antisocial behaviour published between 2012 and 2022.

Authors	Year	Variables	Results
Danioni, Kavussanu, Regalia and Barni [72]	2021	Collective moral disengagement Motivational climate Antisocial behaviour	Collective moral disengagement significantly predicts antisocial behaviour towards teammates and opponents, with the moderation of a performance-oriented climate.
Boardley, Matosic and Bruner [73]	2020	Moral disengagement Antisocial behaviour	Earlier antisocial behaviour was a strong positive predictor of later antisocial behaviour, and earlier moral development was related to later moral development. Moreover, moral development predicted longitudinal changes in antisocial behaviour towards opponents.
Güvendi and Işım Türksoy [74]	2019	Moral disengagement Aggression Fight sports	Youth playing contact sports tended to report higher levels of moral disengagement than athletes in non-contact sports and a higher perception of legitimacy of aggressive behaviour in competition. Younger athletes (17–18 years) tended to report higher levels of moral disengagement than older athletes.
Jones, Woodman, Barlow and Roberts [37]	2017	Narcissism Moral disengagement Motivational climate Social desirability Sex Sport type	Narcissism predicts antisocial behaviour via moral disengagement that remained significant when controlling for motivational climate, social desirability, sex, and type of sport.
Hodge and Gucciardi [75]	2015	Antisocial behaviour Prosocial behaviour Motivational climate Basic psychological needs Moral disengagement	Coach and teammate autonomy-supportive climates have significant direct relations with need satisfaction and prosocial behaviours. Coach and teammate controlling climates significantly relate with antisocial behaviours. Need satisfaction is both directly and indirectly linked with both prosocial and antisocial behaviours. Moral disengagement is directly and indirectly related with antisocial behaviours.
Tsai, Wang and Lo [15]	2014	Locus of control Moral disengagement Rule transgression	An external locus of control is associated with higher levels of moral disengagement compared to the internal locus of control. Moral disengagement is highly correlated with rule transgression among athletes.
Stanger, Kavussanu, Boardley and Ring [76]	2013	Moral disengagement Negative emotion Antisocial Behaviour	The relationship between moral disengagement and antisocial behaviour is partially mediated by anticipated guilt. Attribution of blame reduces negative emotional reactions to antisocial behaviour and increases reported likelihood to act antisocially; this relation is mediated by anticipated guilt.
Šukys [77]	2013	Cheating Moral disengagement Personal Factors	Moral disengagement is a predictor of the general justification of cheating in sports. Deceptive actions associated with athletes’ manipulation of the rules of the sport contest were more justified by athletes with more experience in sports. Deceptive actions associated with athletes’ manipulation of the results of the sport contest were justified to a greater extent by younger athletes.
Bruner, Boardley and Cote [78]	2012	Social Identity (ingroup ties, ingroup affect) Moral disengagement Antisocial behaviour Prosocial behaviour	Overall ingroup affect has a positive effect on prosocial behaviour towards teammates and negative effects on antisocial behaviour towards teammates and opponents; all effects are mediated by moral disengagement. Overall ingroup ties have a positive effect on prosocial behaviour towards teammates and antisocial behaviour towards teammates and opponents; no effects of ingroup ties on the three types of behaviour are mediated by moral disengagement. Finally, neither dimension of social identity influenced prosocial behaviour towards opponents.

## Data Availability

Not applicable.
